# Finding the “Heart” in the Green: Conducting a Bibliometric Analysis to Emphasize the Need for Connecting Emotions with Biophilic Urban Planning

**DOI:** 10.3390/ijerph18189435

**Published:** 2021-09-07

**Authors:** Christopher Tirri, Hunter Swanson, Mahbubur Meenar

**Affiliations:** Community Planning + Visualization Lab, Department of Geography, Planning and Sustainability, School of Earth and Environment, Rowan University, Glassboro, NJ 08028, USA; swansonh9@students.rowan.edu (H.S.); meenar@rowan.edu (M.M.)

**Keywords:** biophilic urban planning, emotion, sentiment, green spaces, cities, environmental benefits, green gentrification, VOSviewer

## Abstract

Although there is a robust body of literature exploring the relationship between biophilic urban planning (BUP) and public health and well-being, there is a dearth of scholarship on the emotional components of BUP. It is crucial to understand these sentiment-related elements, so planners can assign “human value” to green spaces as a strategy for emphasizing the need to thoughtfully implement and properly maintain them in urban environments. Furthermore, humans’ emotional experiences with green spaces may also reveal hidden or unexpected functions of those spaces. To confirm this lack of emphasis on emotions in BUP, we used Scopus to conduct a bibliometric analysis on relevant literature published within the last twenty years (2001–2021), ultimately collecting 589 relevant peer-reviewed articles. We then utilized VOSviewer (Centre for Science and Technology Studies, Leiden University, The Netherlands) to visualize our results and identify thematic, geographic, authorship/co-authorship, publication, and temporal trends. “Green space” appeared as our most frequently occurring keyword and scholars affiliated with institutions located within the United States, the United Kingdom and China were the top producers of relevant results. Our authorship analysis resulted in 67 different clusters and three major but isolated networks. *Urban Forestry and Urban Greening* was the most prevalent source of publication and 2019–2021 was the most prolific period of activity to date. While the goal of our review is to underscore the dearth of controlled, interdisciplinary research on the emotional components of BUP, we also uncovered additional key gaps in scholarship that could promote future avenues of inquiry. First, by focusing on the emotional value of green spaces, practitioners can ascribe them an intangible “human value” that could, in turn, generate more community-focused designs that provide access across socioeconomic, racial and age brackets. Second, an increase in scholarly representation from developing countries could help address the “human value” of green spaces not simply as a “first-world” phenomenon. Finally, a global focus on the emotional, human connections to green spaces may help scholars and practitioners alike mitigate the growing trend of green gentrification.

## 1. Introduction

Since Edward O. Wilson first popularized the concept in his seminal text *Biophilia* (1984), biophilia has gradually gained traction in urban planning vernacular [1], as has awareness of the importance of the connections among nature, people, emotions, and the built environment. The simple presence of nature, though, is insufficient to achieve what Beatley [2] refers to as “biophilic urbanism”, which requires a thoughtful, deliberate incorporation of green elements into the process of urban planning to enhance the ways residents feel about and because of exposure to nature [3]. What makes a city and, by extension, urban planning truly biophilic is the promotion of an active relationship between residents and green spaces where emotional connections and resiliency are at the forefront [4]. Biophilic principles must thus be implemented at various scales (e.g., building, street, block, neighborhood) [5], must be interconnected, ongoing and sustainable [6], and must help create cities that “protect distant nature as well as nature within their borders” ([7], p. 3). What we then refer to as biophilic urban planning (BUP) encompasses multiple types of blue–green infrastructure (BGI), ranging from large urban parks and smaller pocket parks to green stormwater infrastructure projects (e.g., rain gardens, green roofs, community gardens) and local bodies of water, such as lakes, rivers, ponds, and fountains.

The benefits of BUP are well documented, with most scholars reaffirming biophilia’s restorative potential for human health, physical well-being, and psychological wellness [4,5,6,7,8]. Biophilic urbanism also has invaluable benefits for the social functioning of cities and their residents, in that it encourages social activity, interaction and participation [4,9], as well as more profound qualities, such as empowerment, inclusion, equity, and pride [10]. It is the social aspects of sustainability that Totaforti [11] laments have been historically absent from conversations surrounding urban planning, thereby underscoring the importance of human–nature coexistence that biophilic urbanism espouses. Biophilic urbanism thus calls for a revised approach to green spaces no longer as a luxury but rather as a basic life necessity [12,13].

In recent years, there has been a spike in published articles performing reviews or meta-analyses on the extant literature related to green spaces and BGI, although many of those articles tend not to position BGI as one of many practices that fall under the larger umbrella of BUP. Those articles also tend to examine the relationship between green spaces or ecosystem services and the following themes in isolation: public health [14], human well-being [15,16], or urban sustainability [17]. Based on our searches on Scopus, there are few, if any, studies that distinctly utilize the overarching philosophy of biophilia or BUP and connect it with human emotions. Our aim with this review, then, is to trace the contours of the academic literature on biophilic urban planning to identify which themes authors most commonly discuss, how often they discuss them and to argue in favor of developing a deeper, critical understanding of the emotional and sentimental elements inherent in BUP. Because many of the benefits green spaces afford are intangible in nature, it is difficult for researchers, planners, politicians, and the public to assign those spaces a specific economic value [18,19,20]. By focusing on humans’ emotional experiences with, in, and around green spaces, researchers can begin to assign such spaces a “human value” to further highlight their benefits [21] and to endorse their implementation and maintenance within urban environments [20]. Such studies may also reveal hidden or unexpected functions of green spaces and how each of those functions variably impacts users’ emotional experiences [22].

Tracing and synthesizing such a complex network of themes and debates within the study of BUP requires a specific research-oriented approach. As Hood and Wilson [23] highlight, three approaches for citation analysis exist, although their definitions and intentions tend to overlap. The oldest of these approaches is the bibliometric analysis, which involves the quantitative study of the written/published communications within a given discipline. The goal of a bibliometric analysis is to trace, illustrate and explain the evolution of the scholarship within that discipline to identify gaps in research [23]. The second approach, the scientometric analysis, is very closely related to a bibliometric one because of its focus on literature. Its main difference, however, lies in its measurement and analysis of the socio-political implications of the research within science and technology, the practices of researchers within those fields and their real-world influences in the government and the economy [23]. The final, newest, and most general approach is the infometric analysis and it “covers the empirical studies of literature and documents, as well as theoretical studies of the mathematical properties of the laws and distributions that have been discovered” ([23], p. 300). Its distinguishing feature is its inclusion of communications that non-scholarly communities have generated and shared.

Given our aim to determine the extent to which scholars focus on the connection between BUP and emotions, a bibliometric analysis—or a “scoping review” as scholars in other fields may refer to it [24]—stands as the most useful approach, because its emphasis on citations helps reveal networks among relevant authors and, thereby, an image of the larger academic conversation across continents and time [25]. Nalau and Verrall [26] offer a similar defense of bibliometric analyses, touting their use of “statistical analysis of publication metadata” that enables them to “assess much larger literature sets” (p. 3). Historically, these analyses have heavily relied on Web of Science (WoS) to gather relevant publications; however, Scopus has become another invaluable source for research since its creation in 2004 because of its access to more than 20,000 journals (compared to approximately 12,000 in WoS) [25], more than 4,000 of which are fully open access [27]. Although Mongeon and Paul-Hus [28] remind researchers of Scopus’ limited inclusion of publications within the Arts and Humanities, they nonetheless commend its multidisciplinary database for supporting rhetorical and visual content analyses across a wide range of source types, including books, book chapters, reviews, conference papers, and articles, among others. For the purposes of our review, we limited our searches to English-language, peer-reviewed journal articles to produce a more manageable collection of results that would help us identify not only historical and current trends within the extant planning scholarship but also potential avenues for future research that would help propel the field into more human-focused directions.

Our review begins with an overview of our methodology, wherein we discuss how we determined the parameters of our initial data collection, how we refined our searches and our subsequent results and how we visualized our data in VOSviewer. Next, we offer a hybrid section that covers both our results and our discussion of them. We examine our results in five distinct subsections: keyword themes, authorship/co-authorship networks, geographic coverage, publication distribution and temporal trends. In each subsection, we also offer an analysis of each set of results that includes rationales and potential implications for the discipline. Our results section concludes with a succinct content analysis of both the key articles we identified within our initial results and those we intentionally added to support our thematic analyses. Ultimately, we reflect on the gaps in the research related to the intersection of BUP and emotion, with an emphasis on encouraging interdisciplinary collaboration, a more sustained examination of how and why BUP influences human emotions, and a continued focus on the growing concept of green gentrification.

## 2. Materials and Methods

### 2.1. Determining Search Terms and Defining Parameters

The team developed our approach during weekly virtual meetings based on initial conceptualizations of this review, balancing our two key concepts of biophilic urban planning and green infrastructure with the themes of emotion, equity, mental health, and well-being. We then conducted a test search on Scopus using a combination of the terms (“green infrastructure” + “emotion”). This initial search yielded results that inspired the team to modify and expand our list of themes to include the following: emotion, equity, well-being/wellbeing (to accommodate for spelling variations between American and British English), identity, happiness, and perception. Matsler et al. [29] followed a similar methodology when determining which search terms to use, in that they identified recurring terms within their initial collection of review papers, “rather than choosing alternative terms *a priori*” (p. 2). Based on these early search results, the team also agreed to expand our list of key concepts to include biophilic urbanism/urban planning, green infrastructure, green space, and parks.

### 2.2. Collecting and Refining Data

With our final lists of key concepts and themes, we established a streamlined search process for the first and second authors to begin gathering results, such as those Kandel et al. [30] and Nalau and Verrall [26] utilized in their respective bibliometric analyses. For each of our five key concepts—biophilic urban planning, green infrastructure, green space, urban farms, and parks—we conducted seven separate searches so that we paired each concept with the following six themes (three themes per researching author): “emotion”, “equity”, “well-being/wellbeing”, “identity”, “happiness”, and “perception”. Upon entering the search terms, we refined our results by language (English-language only) and document type (articles only). If a certain combination of terms yielded an excess of 2000 results (as did our search for “parks” + “perception”, for example), or if either researching author experienced any significant divergences, all three authors collaborated to further refine the search by subject area to eliminate non-essential or unrelated subjects (e.g., pharmacology, business/management/accounting, and dentistry), resulting in an initial total of 8169 articles. We then recorded the total number of initial results (8169). We manually clicked through that total number of results to identify potentially related articles to use as part of our literature review and bibliometric analysis. To identify related articles, we gauged applicability based on titles and abstracts (if provided) prior to marking any to include in our “saved lists” for each of our searches and tallying the total number of potentially related results (1162).

Although we primarily relied on keywords during our initial collection process, we refined our results in stages using increasingly deeper methods of content and rhetorical analysis. First, we manually clicked through our results and easily filtered out unrelated articles based on their titles, which helped reduce our number of “saved” items from 8169 to 1162. Next, we closely read the abstract for each article to eliminate sources that either had only a tangential relationship to the topic or had misleading titles that bore no similarity to the topic whatsoever. If a certain article did not have an abstract at all, or if the abstract provided left us with lingering questions, we selectively read the content of the article to determine its applicability. In the end, our final “saved list” dropped from 1162 to 857.

### 2.3. Exporting Data and Determining “Best-Fit” Candidates

The final step in working with our newly refined data was to export it from our individual Scopus saved lists and to build a composite Excel spreadsheet so we could eliminate duplicates, any non-article sources that our initial searches did not filter out and any articles published prior to 2001 (a 20-year time frame). This process brought our final number of articles from 857 to 589, a full list of which is available upon request. We worked with this condensed list to determine candidates for our “best-fit” list that would form the basis of our content analysis. We determined candidates based on the following considerations: whether articles initially had duplicates (assuming repetition in multiple searches indicates a strong relationship to the topic); how many times each article was cited by other publications (data Scopus automatically provides); content redundancy and uniqueness of perspective; variety in source of publication. Once we curated a working list of 29 “best-fit” candidates, we supplemented it with additional research to support the various threads that emerged as we drafted our content analysis, rounding out the total to an even 40. For these supplemental sources, we determined their applicability based on currency of research, strength of connection to a particular thread, and the foundational impact in the field.

### 2.4. Importing and Visualizing the Data in VOSviewer

To visualize our data, we used VOSviewer (version 1.6.16), an application that creates networks of authors and keywords based on their co-occurrences, which has been used for similar reports performing meta-analyses of published literature on specific topics [26,30]. To do so, we first had to export our final list of articles as a .csv file, which also allowed us to preserve basic citation, keyword, and abstract information. We compiled all our articles into a single saved list on Scopus, then exported it as a .csv file with all available information on the categories selected before uploading the file into VOSviewer for visualization and analysis.

## 3. Results and Discussion

The graphics produced in VOSviewer analyze bibliometric data by identifying how certain items (e.g., keywords and author names) are related to one another, typically using links combining related items. The sizes of the links vary based on their relative strength, which could indicate different qualities of the data, depending on which item is under analysis. For example, when observing co-occurrence of keywords, the link strength would indicate the number of publications in which two words have occurred. For the co-authorship links, the strength would depend on the number of publications two researchers have co-authored. Closely linked sets of items create color-coded clusters and connections among these clusters form networks. Finally, the size of each node—a circular placeholder for a label for whatever attribute is under analysis—is weighted depending on the number of documents, citations, or occurrences.

### 3.1. Thematic Networks

**Figure 1:**
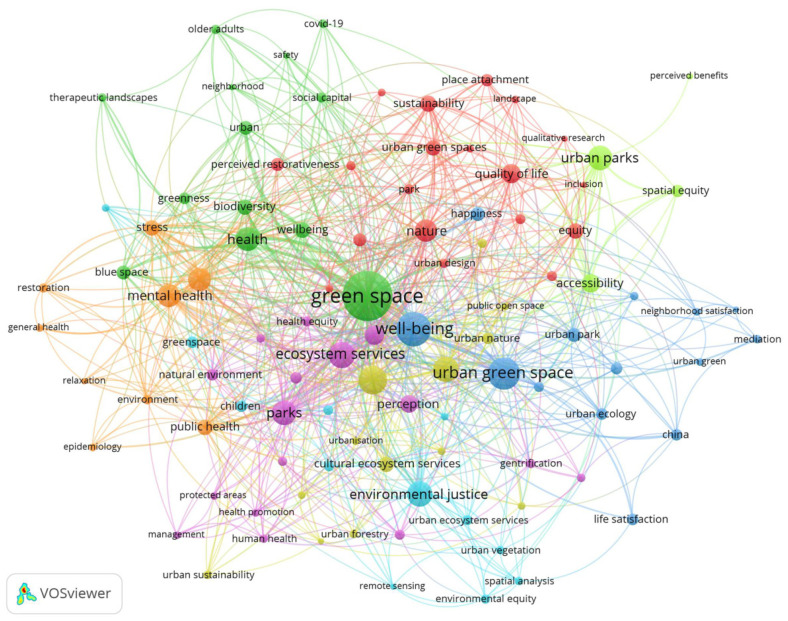
Thematic networks based on the 100 most common keywords from our research.

VOSviewer identified a total of 1588 keywords from our saved lists. Out of this total, however, 1261 keywords occurred only once, such as “cultural services”, “forests”, “natural spaces”, and “community health”. These single-use keywords do not appear in our map because we opted to illustrate only the 100 most frequently occurring keywords for visual clarity, in that the less frequent the term, the smaller the node, most of which are not easily visible on the VOSviewer website or at all (Figure 1). Our most frequently occurring keyword, “green space”, accounted for approximately 6% of the total keywords (n = 97), while the second most frequently occurring keyword, “well-being”, accounted for slightly over 3% (n = 52). Using the clusters VOSviewer generated based on our keywords, we identified eight major research themes: “green space”, “well-being”, “ecosystem services”, “urban planning”, “physical activity”, “environmental justice”, “nature” and “urban parks”. These overarching themes contained many of our more frequently occurring keywords, such as “health”, “urban green space”, “parks”, “green infrastructure” and “mental health”, in addition to more equity-minded terms, such as “gentrification”, “environmental/spatial equity”, and “accessibility”.

Our keyword analysis confirms a major trend we uncovered as we completed our initial data collection through Scopus, which is that other scholars and bibliometric analyses have focused mostly on the relationship between green spaces and public well-being [14]. “Perception” appeared seventeenth in our top twenty keywords, with a total of seventeen occurrences, and “equity” placed twenty-fifth overall, with a total of thirteen occurrences. Neither “emotion” (n = 2) nor “biophilic urbanism” (n = 3) penetrated our top 100 results. We thus offer four main indications based on our keyword analysis. First, although the term “biophilia” has origins in the early-1990s and has regained some level of currency in planning, it is perhaps not most scholars’ first choice of terminology when describing the relationship between the natural and human elements of urban planning [3]. Second, publication dates may provide evidence of some discussion of these ideas in the early twenty-first century, while other dates imply a possible resurgence in critical interest in the late-2010s and early-2020s. Third, “perception” and “emotion” are qualitative in nature; therefore, they require significant amounts of time and labor on the part of researchers, as well as a certain level of reliance on self-reported data from participants [12,31,32,33,34]. Indeed, as Bengtsson [35] emphasizes, a qualitative study is both determined and limited by the “financial resources, time and effort the researchers in a study team are able to invest in trying to understand the phenomena under study” (p. 8). The strenuous nature of these studies related to their creation, execution and analysis may explain their scarcity within our results. Finally, issues of equity and accessibility have been persistent subtopics within the scholarship for decades; however, it appears that only in the last seven years have scholars begun to make those issues the main topics of their research [36,37,38,39]. This relatively recent turn in focus may help explain why clusters of equity-related terms appear on the outer edges of the keyword network illustrated in Figure 1 above.

### 3.2. Geographic Networks

**Figure 2:**
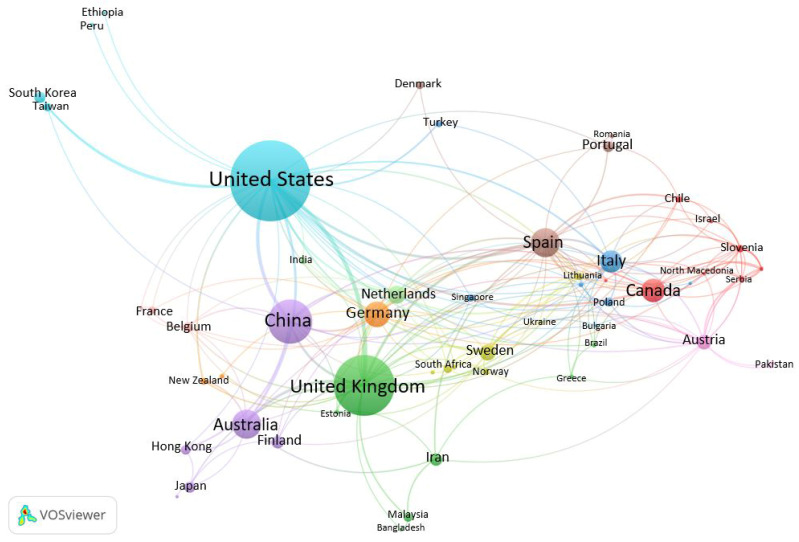
Geographic distribution of relevant publications.

To construct our geographic network analysis, we focused on the countries where authors’ institutions were affiliated, which is what VOSviewer defaults to using, rather than on their geographic areas of study—although the two were not always mutually exclusive. Our distribution analysis revealed that authors were based in 65 different countries, 58 of which were interconnected (Figure 2). Authors affiliated with institutions within the United States, the United Kingdom, and China published the most articles (334 among them), with the United States producing 157 (nearly 27% of our total results), the United Kingdom producing 107 (18% of our total results) and China producing 70 (almost 12% of our total results). Spain, Australia, and Germany produced the second-highest number of articles (n = 114), which was still only about 34% of the total the United States, the United Kingdom, and China produced. The United States had the strongest connections with the United Kingdom, as well as most of the other European countries featured in our saved list. The United Kingdom exhibited similar connections with other European countries. Although China had strong connections with both the United States and the United Kingdom, it featured fewer links to other countries, aside from Australia, Canada, and Germany.

These trends in geographic distribution networks echo prevailing foci on sustainability and the human–nature connection in the planning process within developed countries [15,16,17]. Indeed, Nalau and Verrall [26] postulate that the United States’ increased focus on climate change-related policy during the Obama administration and the United Kingdom’s Climate Impact Program may be major inspirations for these countries’ growing bodies of scholarship. Although some developing countries, such as Iran, Lithuania and Slovenia did produce results (n *=* 13, 7, and 7, respectively), our analysis revealed similar gaps in the literature regarding developing countries that other scholars have highlighted [15,17,40]. Developing countries are more likely to experience rapid, poorly planned, or unplanned urbanization, resulting in highly pronounced environmental degradation and lack of space (both personal and green) [40,41]; however, our analysis seems to reveal a large gap in the literature regarding how those developing countries utilize the principles of BUP.

### 3.3. Authorship/Co-Authorship Networks

**Figure 3:**
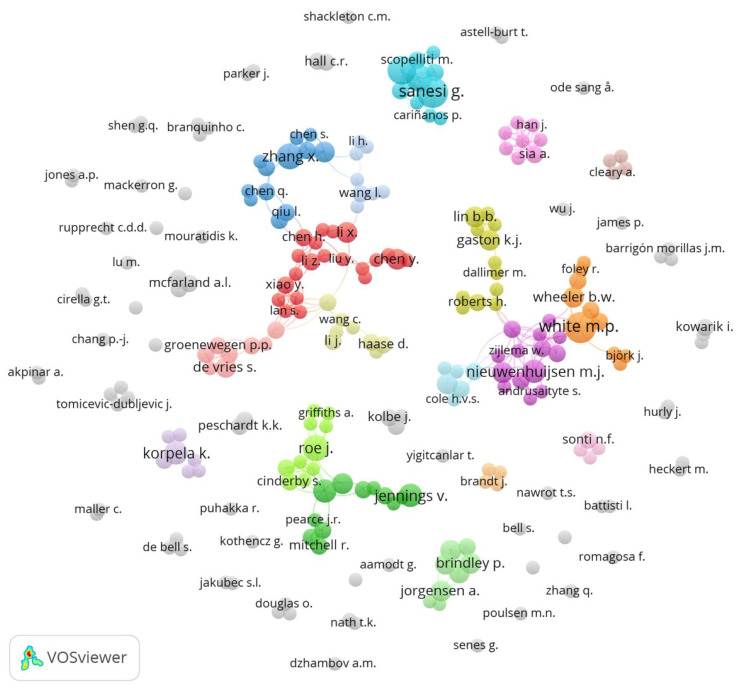
Emerging authorship and co-authorship networks.

We registered a total number of 1913 different authors into VOSviewer from our initial data collection. With a threshold set at the minimum of two documents and at least one citation, our authorship and co-authorship analysis shows that 246 authors collaborated on research exploring the relationship among BGI, access, public health/well-being, and perception, resulting in a total of 67 different clusters of varying sizes (Figure 3).

Of these 67 clusters, there are three main networks that emerge, although they do not connect amongst themselves or with other smaller clusters, suggesting a certain level of scholarly isolation within authorships. Sanesi, G., Carrus, G., and Lafortezza, R. formed the cluster with the largest number of authored and/or co-authored documents (n *=* 21, roughly 31% of the total), with Sansei, G. co-authoring eight articles (about 3% of the total)—our highest individual count among authors. Table 1 captures important statistics for each of our top 10 authors, including the number of articles they each authored or coauthored and the number of times their work was cited by other scholars.

Our authorship and co-authorship analyses are somewhat in line with the results from our prior geographic analysis, in that each of our top three highest producing countries have some level of authorship representation here—two each from both the United States and the United Kingdom, but only one from China. While authors from the United States are more numerous in general, their authorship and co-authorship outputs are fewer in total, with many of these authors only publishing one or two articles, hence their lack of representation in this analysis. Spain ranked in our second-highest geographic group, so its single-author representation here makes sense. Interestingly, Italy has the highest numbers of authors (n *=* 3), despite not ranking in either of our top two tiers in our geographic analysis. Italy’s high numbers are representative of the recent push across the country to address the alarming gaps in national scholarship regarding what Romano et al. [66] call the “urban evolution on the Italian territory” (p. 1). Historically, because there was no authority documenting how land changed hands in Italy, landowners did not keep detailed records about land development over time, which has thus prevented scholars from publishing. Thanks in large part to efforts from the Central Institute of Statistics (Istituto Centrale di Statistica, or ISTAT) and the Higher Institute for Environmental Protection and Research (Istituto Superiore per la Protezione e la Ricerca Ambientale, or ISPRA), Italian scholars now have contemporary methods to collect, synthesize and analyze land-use data for global publication [66].

### 3.4. Publication Networks

**Figure 4:**
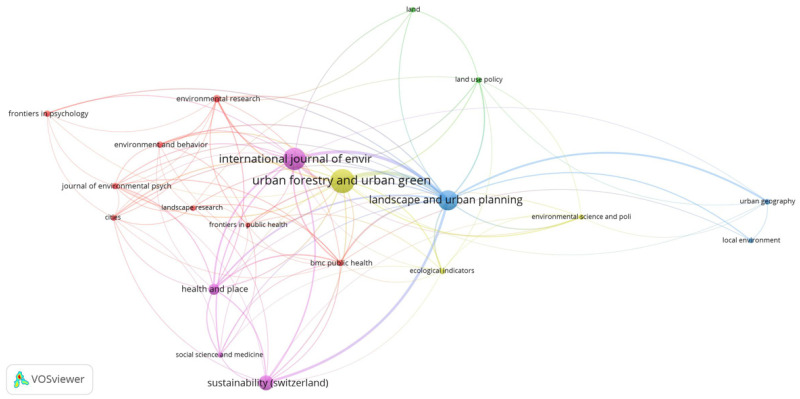
Emerging publication networks of research on the relationship among biophilic urban planning, BGI, emotion, equity, and public well-being.

The articles in our saved list were published by a total of 214 different journals; however, to identify the most relevant and most commonly used/cited journals, we implemented a threshold of a minimum of five documents per journal (Figure 4). *Urban Forestry and Urban Greening, International Journal of Environmental Research*, and *Public Health and Landscape and Urban Planning* were the three most prevalent journals within our saved list, constituting almost 71% of our total results (n *=* 171). *Urban Forestry and Urban Greening* was the most popular journal within our searches, accounting for 11% of the total documents (n *=* 66), followed by the *International Journal of Environmental Research and Public Health* at approximately 10% (n *=* 58), then *Landscape and Urban Planning* at 8% (n *=* 49). However, *Landscape and Urban Planning* yielded the highest number of citations (n *=* 4438), almost three times as many citations as the next two journals (*Urban Forestry and Urban Greening* with 1407 and *International Journal of Environmental Research and Public Health* with 1157).

Our journal coverage analysis indicates that, as expected, most of the relevant literature has been published by journals whose main coverage is urban planning or a closely related subfield (e.g., urban greening, landscape, and sustainability). Although the number of documents published in non-planning journals was generally lower than that of those published in planning journals, it is nonetheless a positive indicator of the field’s growing tendency toward interdisciplinarity. Given the qualitatively rich relationship among biophilic urbanism, planning, emotions, perception and health, an increased co-authorship network of planning, social science, psychology, and health scholars would yield insightful results [7,12,13,52].

### 3.5. Temporal Networks

**Figure 5:**
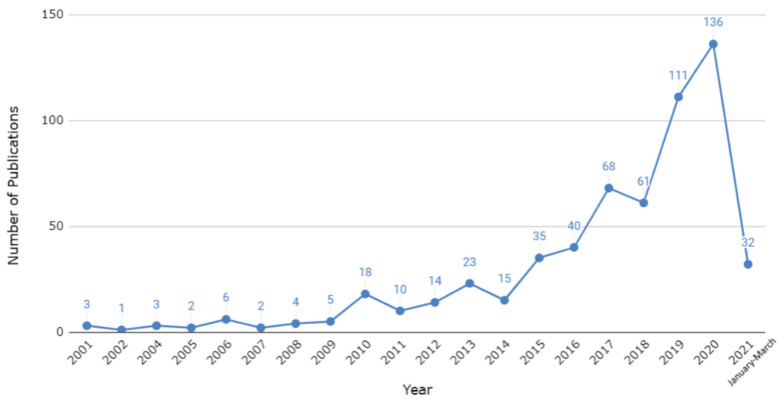
Temporal trends in the publication of relevant research. Note: our data collection for 2021 was completed during March 2021, hence the apparent “drop” in relevant publications.

The increase in scholarship regarding biophilic urban planning, BGI and public health and well-being was minimal during the first decade of the 2000s and scholarship did not consistently grow above thirty-five articles per year until 2015. As we mentioned in Section 2.3 above, we limited our analysis to focus on articles published within the last twenty years (2001–2021) for manageability purposes. Over this twenty-year span, our sample revealed that 589 relevant articles have been published, including thirty-two articles already published in the first three months of 2021 (Figure 5). We observe four general periods of activity based on our results: (i) 2001–2009, where we recorded only twenty-six relevant articles (only slightly more than 4% of our total results); (ii) 2010–2014, where numbers fluctuated inconsistently, resulting in a total of eighty relevant articles (approximately 14% of our total results, with 2011 having the lowest number (n *=* 10) outside of the period between 2001 and 2009); (iii) 2015–2018, where results consistently increased every year, with a total of 204 relevant articles (nearly 35% of our total results); (iv) 2019–2021, where results have reached above 100 each year, totaling 279 relevant articles (47% of our total results), with 2021 poised to be the most prolific year yet (n *=* 32 at the time of writing, or 5% of our total results). To date, 2020 has the highest number of relevant results, with 136 (23% of our total results).

Aside from an increased attention to issues of environmental justice and sustainability, as well as rapid unplanned urbanization, on a global scale, three specific events may help explain the shifts in temporal trends detailed above. First, according to the American Planning Association’s [67] Planning History Timeline, 2011 marked the first time the United States Census Bureau reported that cities were growing faster than their suburban counterparts. Although that trend decelerated by 2016 and even more so because of the COVID-19 pandemic, it would have nonetheless inspired urban planners to heavily revise their understandings of and approaches to the concept of “the city”. Second, the United Nations [68] published its 17 Sustainable Development Goals (SDGs) in 2015 as part of its 2030 Agenda for Sustainable Development, which have formed the basis of much of the theory and practice of urban planning across the globe in the years since. These goals rightfully call for global collaboration among developed and developing countries to focus not just on preserving the natural environment but also on empowering the people within it [68]. Finally, the Paris Agreement on Climate Change was ratified in December 2015, marking another major step toward global efforts to reverse humans’ negative influence on the natural environment. Together, these events have propelled the discipline forward to offer more critical evaluations of how humans interact with their environment and how the environment, in turn, influences human behavior, emotion and health.

By contrast, a more logistical explanation for the temporal trends we observed may simply be the growth in the number of journals that Scopus indexes or the global growth in scholarship concerning issues of environmental justice and sustainability, higher populations in cities and global planning efforts that foster resilience, human empowerment, and the mitigation of climate change.

### 3.6. Content Analysis

While conducting our content analysis, we identified the following major theoretical threads: the personal, socio-economic, and environmental benefits of BUP; issues of access to and equity in BUP; the dangers of green gentrification; how the COVID-19 pandemic further amplified those issues; challenges to current approach to BUP; the lack of scholarly inquiry on the connection between human emotions and BUP.

Scholars often defend the positive effects BUP can have on self-reported human health and well-being [12,31,32,33,34]. Personal benefits include positive correlations with life satisfaction and quality of life [32,33,52,69], the reduction of stress and mental fatigue [12,52,70], a boost in identity and sense of place [34], and a greater appreciation for nature [31]. Socioeconomic benefits include a reduction in crime [33] and possible economic stimulation due to increased tourism and property values [34], although some authors warn of the “elite enclaves of environmental privilege” such stimulation can create [36]. Finally, environmental benefits occur through the reduction of polluting or fragmenting grey infrastructure [34], the filtration of air [70], the reduction of the urban heat island effect [70,71], and the creation of natural green stormwater infrastructure that eases the burden on man-made stormwater systems [71,72].

Many of these benefits, though, especially the personal ones, hinge upon high, consistent levels of access to green spaces [71]. While White et al. [52] claim “green spaces such as parks are accessible to all” (p. 926), most of the relevant scholarship disagrees with such a claim based on mounting evidence of unequal distribution of/access to green space due to historical environmental injustices that disproportionately affect lower-income, minority areas [10,69,73,74,75]. Research shows green stormwater infrastructure projects in some cities are also inequitably distributed [76] or constructed without much consideration of social factors [77,78]. As Wright Wendel et al. [79] highlight, such inequitable distribution of and access to green spaces is not unique to developed nations; indeed, it is also present, if not more pronounced, in rapidly developing nations.

Although increasing access to green spaces across the board may seem an obvious solution, scholars warn of the dangers of green gentrification [36,37,38,39], which occurs when urban planning fails to account for the long-term, unintended consequences of providing greater access to BGI in less-affluent areas, namely the forced displacement and alienation of at-risk residents. Wolch et al. [39] offer the “just green enough” strategy as a remedy for green gentrification because it privileges community-based design over market-driven design, when it comes to providing better access to BGI, while other scholars emphasize the need to revise the actual design of urban parks [80], individual perceptions of those parks’ safety and their level of maintenance [59] to more broadly address diversity-related concerns within the environmental justice movement [32].

The COVID-19 pandemic further complicated the debate over access to and use of BGI [81,82,83]. Pouso et al. [50] insightfully analyze the relationship between the loss of the home environment’s restorative effects due to stay-at-home mandates and the increased use of BGI to compensate for that loss. In line with much of the non-pandemic literature on the restorative effects of nature, pandemic-era scholars report positive correlations among views of and access to BGI and physical and mental wellness [50,81,83,84], as well as “social cohesion and spiritual wellness” ([81], p. 553). Levels of usage varied on local, regional, and national or global scales based on individual countries’ social distancing protocols [81,83,85], although demand for green spaces [81] and an understanding of the role they play in environmental consciousness [83] increased across scales. COVID-19 also amplified the systemic issues behind access to BGI, with authors such as Uchiyama and Kohsaka [82] highlighting how socioeconomics, education and land-use patterns have had a profound impact on underprivileged residents’ access to BGI, as well as the types of BGI they sought out, during quarantine.

Even scholars who recognize the value of green spaces nonetheless question how many of these spaces a single urban environment needs [10,70], how they should be integrated with urban environments [41,78,86] and what specific role they play in potentially enhancing human health and well-being [69]. Others explore how BGI benefits users at various stages of the life cycle [87], as well as the strength of the correlation between nature and human health based on either the amount of/proximity to green spaces [13] or the level of biodiversity they promote [3,31]. Others still question the methods previous researchers have used to identify potentially inaccurate causal relationships between green spaces and health, offering instead new frameworks to uncover more concrete and accurate connections [88].

For planning to be considered truly biophilic, it must balance the creation, renovation, and maintenance of various types of BGI with a deliberate acknowledgment of the people who do/do not, might/might not and can/cannot use those spaces. As the COVID-19 pandemic has revealed, humans rely on green spaces for their physical and mental health and those spaces, in turn, offer well-documented environmental benefits in both the short- and long-term. Scholars have only relatively recently begun to identify the social impacts of BGI through the lens of green gentrification, a rapidly growing field of inquiry dedicated to investigating how planners can address issues of environmental and social justice. Fewer scholars, though, seem to have explored the emotional aspects of the relationship between humans and BGI, which we believe would yield invaluable insights for planners when conceptualizing, refining, and redeveloping the kinds of green spaces residents both want and need in their localities. One notable recent study examined the impact of “greening” vacant urban land, revealing that residents’ self-reported feelings of depression and worthlessness significantly decreased in those living close to newly-greened lots, compared to those living around vacant lots that remained blighted [89].

The few scholars who have conducted large-scale sentiment analyses have primarily leveraged the social media platform Twitter and used text mining methodologies to quickly collect and analyze pre-existing user-generated data [20,21,22]. Platforms such as Twitter allow researchers to gain insight into individuals’ emotional reactions to green spaces as they experience those spaces in “real” time [20,22]. Each study, however, highlights critical limitations to applying controlled methodologies to analyzing uncontrolled data. Although manual methods may provide the most accurate observations, they are time-consuming and often impossible because of the amount of data and automated or semi-automated methods rely on inaccurate algorithms [20]. Tweets and other posts may also contain highly subjective, multiple, conflicting, or even non-alphabetic emotions that resist easy classification [20]. Finally, Twitter users may only represent limited demographics of platform subscribers and/or green space visitors, with adult users taking precedence over younger, elderly, and underrepresented users [21,22]. While controlled qualitative studies are potentially more time-, labor-, and cost-intensive, it is clear simply lifting readily-available user data from social media platforms is not a viable, long-term approach for planners who hope to develop a more accurate body of results to confirm or challenge these Twitter-based studies.

### 3.7. Limitations

During our data collection, we encountered a few crucial limitations. As we discussed in our Materials and Methods Section, some of our initial searches returned 5000 or more results, which far exceeded our team’s capacity. To obtain a more manageable but nonetheless representative sample of relevant articles, we thus had to utilize some combination of Scopus’ numerous filters (e.g., date of publication, genre of publication and subject area). Another team of researchers, regardless of the number of members, may generate a different set of relevant results based on their unique combinations of filters. Additionally, Scopus primarily collects publications in English, so relevant articles in other languages may very well exist outside the scope of our research. Finally, our methodology heavily relied on Scopus’ use of keywords and known phrases to identify potentially relevant articles. Although these keywords and phrases helped streamline the process, they realistically cannot provide a comprehensive picture of the extant literature. Using VOSviewer to visualize our results generated two significant limitations. First, we needed to reduce the number of results under visualization so that labels would be legible. This was especially difficult during our keyword analysis due to the sheer volume of keywords. The resulting thematic networks are therefore slightly less robust or interconnected than they may have been if we were able to display a greater number of keywords. Second, the VOSviewer system does not offer an option to analyze either titles or full abstracts, which meant we had to conduct our own manual title and abstract analyses when attempting to locate relevant results.

## 4. Conclusions

In this review, we utilized Scopus to analyze the current state of the body of research concerning the relationship among BUP, BGI, emotion, equity, and public well-being. We encourage future researchers to perform similar analyses using other databases, such as PUBMED or WoS, to identify patterns or gaps in results. Our VOSviewer-based bibliometric analysis of twenty years’ worth of peer-reviewed, English-language journal articles examined emerging keyword/thematic trends, geographic representation, authorship/co-authorship networks, publication coverage and temporal distribution. As we anticipated, the most common keywords among our results were “green space” and “well-being”, with six additional research themes and related concepts emerging. Geographic representation continues to favor developed countries, such as the United States, United Kingdom, and China, while developing countries are only beginning to penetrate the field. Co-authorship networks tended to be very insular or limited, resulting in at least seven major networks, none of which connected with another. Three journals were most prominent among our results—*Urban Forestry and Urban Greening, International Journal of Environmental Research and Public Health*, and *Landscape and Urban Planning*—while scholarship, overall, has greatly increased over the past decade.

Our review and subsequent observations contribute to the existing field of research in three major ways. First, we observe that, while the well-studied connection between BGI and public health and well-being has revealed important, real-world consequences for BUP in terms of its ability to influence practice and policy, future studies need to extend their scope to include the value of creating green spaces that cultivate positive emotional experiences. Second, the COVID-19 pandemic has dramatically altered the way we perceive, design, and reside in urban environments, placing access to BGI at the forefront of the critical conversation and reiterating the “human value” of green spaces. Finally, we identify two additional avenues for future research, (1) increasing conversation about the role of BUP projects within dense cities in developing countries and (2) developing a “just green enough” approach to BUP that utilizes research on humans’ emotional connections to green spaces to help mitigate the (sometimes unintentional) harmful consequences of green gentrification.

As scholars have continued to extol the positive connection between BGI and public health and well-being, urban planners have increasingly taken their findings into consideration, particularly evident in the proliferation of “compact cities” characterized by highly dense and mixed-used patterns [10]. Scholars’ findings have also proven that it is not just a matter of increasing the quantity of green spaces but also a matter of enhancing the quality of those spaces through a variety of types and uses, thoughtful design and size, the promotion of interpersonal engagement and the cultivation of positive emotional experiences. In other words, planners must produce responsive designs that promote usage across socioeconomic levels, racial/ethnic backgrounds, and age, as well as ones that address issues related to social and environmental justice. Beyond responsive design, scholars emphasize the importance of approaching BGI from a flexible, process-oriented perspective if its implementation is to be a long-term success [41].

The COVID-19 pandemic has further underscored the importance of a flexible approach to urban planning, biophilic or otherwise. Considering lockdowns and work-from-home mandates across the globe, urban environments figuratively shrank as residents were unable to utilize many of the contemporary amenities that may have originally attracted them to city living. Yet, those environments also shrank in terms of population, as large numbers of upwardly mobile residents fled back to the suburbs [90]. For those residents who chose to remain in the city, or were unable to relocate, urban green spaces became a highly sought-after respite from quarantine’s psychological burdens [81,84]. Demand for these spaces reinvigorated the importance of their equitable distribution, especially in urban neighborhoods that have historically faced environmental injustices. Such reinvigoration paralleled scholars’ findings about the impact socioeconomics, education and land-use patterns have had on those neighborhoods’ residents’ access to BGI during quarantine [82]. Relatedly, the gray literature has repopularized the concept of the 15- or 20-min neighborhood as a possible solution to issues of green space equity [91], and we anticipate the concept once again making its way into academic scholarship. Ultimately, quarantine served as a much-needed reminder that residents’ emotional connections with BGI are equally as important as the mental, physical, and environmental benefits of those projects.

The results of our geographic network analysis revealed scholars affiliated with institutions located within the United States, the United Kingdom, and China as the most prolific publishers for relevant scholarship within our bibliometric analysis, with only a small handful of other developed nations rating below them. This geographic network analysis also revealed a striking need for future research on developing countries’ individual approaches to BGI, authored by scholars from within those countries. What further complicates this lack of planning research on and from developing countries is Chimhowu et al.’s [92] observation that there is a profound, renewed global interest in comprehensive national planning, evidenced by the fact that the number of countries with a national development plan has more than doubled from 62 in 2006 to 132 in 2018. They describe this increase “as a catalyst for achieving Agenda 2030 for Sustainable Development” ([92], p. 76) the goal of the 17 Sustainable Development Goals the United Nations codified in 2015. If these goals are genuinely generating a renewed interest in planning across the globe, then planning scholarship needs to reflect it.

Finally, planners and related professionals need to remain cognizant of the consequences—intended or otherwise—of implementing green initiatives within BUP. As some scholars have highlighted, large-scale BGI projects are not always the most effective solutions, because they have the dangerous potential to displace the very communities they seek to improve [36,37,38,39], thereby forsaking the “human value” of the spaces they intend to design or promote. Thus, plans at the continental, national and regional levels must be coordinated to focus on making communities “just green enough” so that socioeconomic development occurs in a thoughtful, controlled way, rather than in a way that results in widespread green gentrification [39].

## Figures and Tables

**Table 1:** Top 10 authors’ numbers of relevant publications and times cited.

Name of Author (Last, F.)	Number of Relevant Articles	Number of Times Cited
Sanesi, G. [31,32,42,43,44,45,46,47]	8	721
White, M.P. [48,49,50,51,52,53,54,55]	8	666
Carrus, G. [31,42,43,44,45,46,47]	7	705
Lafortezza, R. [31,32,42,43,44,46]	6	704
Roe, J. [56,57,58,59,60,61]	6	269
Brindley, P. [62,63,64,65]	4	27
McEwan, K. [62,63,64,65]	4	27
Richardson, M. [62,63,64,65]	4	27
Sheffield, D. [62,63,64,65]	4	27
Colangelo, D. [31,42,47]	3	370
